# The world is coming to an end! COVID-19, depression, and anxiety among adolescents in Malawi

**DOI:** 10.3389/fpsyt.2022.1024793

**Published:** 2023-01-05

**Authors:** Chilungamo Mmanga, Yamikani Ndasauka, Jimmy Kainja, Fiskani Kondowe, Martina Mchenga, Limbika Maliwichi, Simunye Nyamali

**Affiliations:** ^1^Kamuzu College of Nursing, University of Malawi, Zomba, Malawi; ^2^Department of Philosophy, University of Malawi, Zomba, Malawi; ^3^Department of Media and Communication Studies, University of Cape Town, Cape Town, South Africa; ^4^Department of Mathematical Sciences, University of East Anglia, Norwich, United Kingdom

**Keywords:** mental health, adolescents, COVID-19, Malawi, depression, anxiety

## Abstract

**Introduction:**

This paper assessed the effects of Covid-19 on adolescent mental health in Malawi. There is minimal research on adolescent mental health in Africa, Malawi in particular. The study shows a link between the pandemic and mental health. Some factors that may have contributed to this link include; Covid-19 preventive measures, media exposure and the increase in unemployment.

**Methods:**

The study used a mixed methods approach, quantitative and qualitative methods. It was conducted in Malawi's four districts (Blantyre, Mangochi, Lilongwe and Karonga).

**Results:**

Overall 22%, 21%, and 23% of the respondents had depression, anxiety and post-traumatic stress disorder, respectively. The Chi-square test showed that significantly more adolescents with secondary education (28%) had anxiety than those with primary education (14%). Further, regression analysis revealed that adolescents with anxiety were 18 [95%CI: 9.34, 35.8] times more likely to have depression compared to those who did not have anxiety. The study found no significant differences in the proportions of adolescents with the three outcomes when comparing different groups within the explanatory variable. The ratio of female and male adolescents with depression and anxiety was the same.

**Discussion:**

The adolescents expressed that Covid-19 affected their social, academic, and financial status. These effects had a significant bearing on their mental health in that they led to depression, anxiety, fear of the unknown, and stress. During the Covid-19 pandemic, adolescents' mental health diminished and posed a considerable risk to productivity of adolescents. As a result, adolescents may not fully realize their potential, form and maintain good relationships, contribute to their community and become resilient. These effects have devastating consequences for this young generation without proper coping strategies.

## Background

The study aims to assess the effects of COVID-19 on mental health among adolescents in Malawi. The COVID-19 pandemic has affected people globally indiscriminately. To take measures against the spread of the virus, governments put restrictions such as restricted movements worldwide. This led to social isolation and adversely affected mental health (1). Everyone was affected by the COVID-19 regulations, especially adolescents. This is because adolescents are at the developmental stage, which relies heavily on peer connections for emotional support and social development (2). Further, these restrictions have been particularly difficult for adolescents, who need peer connections most (3). Adolescence is a crucial transitional stage marked by physical, social, and cognitive development. Hence, the disruptions caused by the pandemic may have had a long-term impact on adolescents' growth and well-being. Evidence shows that half of the mental health conditions begin to manifest at 14 (4). Evidence shows “the potential long-lasting impact of quarantine measures on mental health, which include depression, post-traumatic stress, anger and emotional exhaustion” (1).

In March 2020, the Government of Malawi declared a national disaster in response to COVID-19. The country adopted several measures, including closing schools and universities and limiting church and public gatherings to 50 people only for 1 h or less. The Government also implemented COVID-19 screening at national border posts. In addition, the Ministry of Health encouraged the adoption of protective behaviors such as regular hand washing, physical distancing, using face masks, and working from home. A national lockdown was announced on April 18 2020; however, its implementation was prevented by the Malawi High Court (5). Being landlocked and underdeveloped, the Government of Malawi could hardly sustain lockdown measures. So, even though schools were closed, people were not restricted to their homes, as markets and other public gatherings were still crowded. In addition, there was panic, especially among adolescents, as the Government initiated these measures without massive awareness of the disease to the general public.

On the global scene, studies show that the most prevalent mental health problems associated with COVID-19 are stress, anxiety and Depression (6). Research has found that fear may lead to anxiety and depression. For instance, a study from China indicated that 8.4% of the general population reported severe pressure, while 4.3% reported severe Depression (7). Mental health adversities include being single, unemployed, at risk of health complications, job loss, and financial distress due to the pandemic (8). Besides COVID-19 being a global pandemic and public health crisis, it has severely affected the global economy and financial markets. Specifically, the pandemic has reduced income, increased unemployment, and disrupted transportation, service, and manufacturing industries. These are some consequences of the disease mitigation measures implemented in many countries (9). Women, adolescents and other vulnerable groups may be at the most risk of COVID-19.

The COVID-19 pandemic resulted in fear, anxiety and concern in many nations. Apart from the fear of the pandemic itself, the other sources of worry and concern included; reduced social interaction and support, restrictive public health measures, travel bans, border closures, quarantine measures, and physical distancing that contributed to increased levels of loneliness, anxiety and Depression (10). In an article that systematically reviewed the general population's mental health status, Xiong et al. (11) found that a higher prevalence of adverse psychiatric symptoms had been reported than before the pandemic. The review found that the COVID-19 pandemic represents an unprecedented threat to mental health in high, middle, and low-income countries (11).

World Health Organization estimates that approximately 6–8% of young people live with depression. However, there is limited published literature on the prevalence of mental disorders among adolescents in Malawi and sub-Saharan Africa. Despite the lack of adequate evidence in our setting, some research established that among youth aged 12–24 years of age, there is a 20–25% annual risk of having a mental health diagnosis. Therefore, Matandika et al. (12) studied the prevalence of common mental disorders (CMD) and the factors associated with mental disorders among children and adolescents in Blantyre- Urban, Malawi. They found that the overall prevalence of mental disorders was 5.9% and was higher in males (7.1%) than in females (4.7%). Hence, more research is needed in sub-Saharan Africa to understand the scale of adolescent mental health problems.

Mental health problems are a well-known problem in Malawian society; the problem is primarily understood from cultural and religious beliefs, not as a health condition caused by biological, psychological and social issues. Thus, for many people, mental health is essentially a cultural issue. This is why mental health conditions are not seen as illnesses like malaria or Tuberculosis (13). However, there is currently no consensus on the actual meaning of mental health, although the problem is recognized in the community. Despite the recognition of the problem, those affected by mental health problems do not always come forward for fear of being stigmatized, which makes it difficult to manage mental health timely (13).

Depression and anxiety are some of the mental health problems faced in Malawi. Udedi (14) found a depression prevalence rate of about 30% in attendees of the Matawale Health Center in Zomba, whereas Kauye et al. (15) reported a rate of 19% in attendees of other clinics. In a study of pregnant women and young mothers (many of whom are teenagers), Stewart et al. (16) found rates of depression ranging between 10.7 and 21.1%. Kim et al. (17) report a Depression rate of 20% in adolescents attending HIV/AIDS clinics. These data are similar to those reported in Nigeria (18) and Kenya (19), where in-school adolescent Depression rates are 21.2 and 26.4, respectively (20). In people living with HIV and AIDS (PLWHA), the prevalence of depression has been documented to be as high as double that of the general population. In the few studies in Africa, estimates of the prevalence of Depression in PLWHA range between 12 and 60% (21). One cross-sectional study aimed at estimating the prevalence of depression amongst a sample of HIV-positive adolescents in Malawi showed the results of 21.6% in females and 15.4% in males (17). Another study in Malawi on Adolescents living with HIV (ALHIV) revealed a depression prevalence rate of 18.9% (22).

In terms of training mental health professionals, the colonial Government did not invest in training native Malawian mental health professionals. But when Malawi became independent, it started sending its people to train as psychiatric nurses abroad in Britain. This training has significantly assisted in increasing the number of mental health nurses in Malawi. Later on, St John of God College of Health Sciences was established in 2003 and started offering a University Diploma in Counseling. In 2004, a bachelor's degree in Mental Health-Psychiatric Nursing was introduced to upgrade nurses who wanted to specialize in mental health. In 2008, the college introduced a Bachelor's degree in Clinical Medicine specializing in mental health. In 2004, the Catholic University of Malawi started training social workers at a bachelor's degree level. These are essential human resources for the community mental health care of patients. However, there is still a demand for more trained professionals. According to WHO (23), for every 100,000 people in the country, there are 0.01 psychiatrists, 0.22 mental health nurses and 0.01 social workers (24).

Following this background, the study aimed to address the following research questions; what is the knowledge of mental health and mental health illness among adolescents in Malawi? How did COVID-19 affect the mental health of adolescents? What is the prevalence of depression and anxiety among adolescents during the COVID-19 pandemic?

### Theoretical framework-operant conditioning theory

Operant conditioning is one of the behavioral theories that explain the development and persistence of depressive symptoms as the result of decreased environmental reward, associated reductions in positively reinforced healthy behavior, reinforcement of depressive or passive behaviors, and punishment of healthy behaviors (25, 26). It states that depression is caused by removing positive reinforcement from the environment (25). Certain events, such as losing your job, induce depression because they reduce positive reinforcement from others. Further, this tends to reinforce maladaptive behavior. This eventually alienates even close friends leading to even less support and increasing social isolation and unhappiness.

In addition, if the person lacks social skills or has a very rigid personality structure, they may find it challenging to make the adjustments needed to look for new and alternative sources of reinforcement (25). As such, they get locked into a negative downward spiral. Behavioral avoidance coping occurs when a problem is avoided through participation in alternative activities, temporarily satisfying albeit maladaptive behaviors, or overtly displaying unpleasant emotions' manifestations (27). Considering that COVID-19 is a public health issue, the study thought that the burdens brought about by COVID-19 removed some positive reinforcement in adolescents. Overstressing the negative thoughts related to COVID-19 may have contributed to anxiety and depression. In addition to misinformation being shared on social media, many conspiracies were developed during the pandemic. This brought fear and anxiety among adolescents and the general population. The helplessness associated with contracting the virus left most people worried and depressed. Worry could both exacerbate and be exacerbated by existing anxiety and depressive symptoms (28). In addition, many adolescents used negative coping strategies, including abusing alcohol and other substances. Socially, people were encouraged to practice social distancing. Hence access to social support was not easy for most young people and the general population. Most social gatherings that promote connection and a sense of belongingness were banned. Worse still, they lost their loved ones. These are risk factors for mental health illnesses, including anxiety and depression.

## Methodology

### Study design

This study used a mixed-methods approach, combining qualitative and quantitative data. Data were collected from Blantyre, Mangochi, Lilongwe and Karonga, representing the country's southern, eastern, central and northern regions. Lilongwe and Blantyre were selected because they are major cities in Malawi with a high urban adolescent population and registered high numbers of COVID-19 cases. Mangochi was chosen because it is a lakeshore district, which attracts tourists, and Karonga was selected because it is a border district, which makes it a possible entry point for imported cases.

Qualitative data were collected in all four districts using Focus Group Discussions (FGDs) and Key Informant Interviews (KIIs). Four FGDs (one in each district) discussions were conducted with adolescents. To observe COVID-19 social distancing restrictions, FGDs were limited to 6 people per group and held outside in the open. Participants were recruited through Youth Organizations working in the respective areas. We used convenience sampling to identify these participants: 12 KIIs targeted parents, teachers, community leaders and social workers. In addition, we interviewed a Head Teacher, a community leader, a parent, and a Social Worker in each of the four districts. Again, purposive sampling was used to select the key informants. Qualitative research enabled us to explore the complexity of adolescent behavior and generate a more profound understanding of the COVID-19 pandemic behavior.

Quantitative data was collected from 340 adolescents using structured individual questionnaires. This data was used to explore the relationship between basic demographics and levels of depression and anxiety in the study population. This sample was calculated using the finite population sample size formula (29) and based on the national population of people 10–19 years, as reported in the 2018 Malawi census report. We initially planned to use probabilistic sampling approaches, but convenience sampling was utilized after realizing that probabilistic sampling would be hard to attain. This was partly due to the COVID-19 restrictions put in place during the time of the study. However, our sample is still informative for our study questions, and the results and discussions are mainly based on and strengthened using qualitative data.

The study data was collected from May to June 2021, after the second wave of COVID-19 in Malawi, which was more devastating than the first wave (30).

### Data collection tools

Interview guides were used to conduct KIIs and FGDs. The Key informant interview guides were adapted to the key informant and comprised the following general questions: How has the COVID-19 pandemic affected adolescents? In your observation, has the adolescent felt restless, wound up, or on edge in relation to COVID-19? Has the adolescent displayed any hopelessness or helplessness in their outlook on life? Has the adolescent been showing any lack of energy and an overwhelming feeling of fatigue? FDG guides comprised the following questions; What words or feelings come to mind when you think about the COVID-19 pandemic? How has your life changed due to COVID-19? Have you experienced little interest or pleasure in doing things during the pandemic? Ever since the coming of COVID-19, have you had trouble falling asleep, staying asleep, or sleeping too much?

The study utilized the Generalized Anxiety Disorder (GAD-7) Patient Health Questionnaire (PHQ-9) (31–33) to collect quantitative data. The two questionnaires have been used and validated in various populations as brief screening measures for depression and anxiety. Additionally, the PHQ-9 has been validated in Malawi (14). PHQ-9 has nine items which measure depression on a scale of 1–27. Scores of 5, 10, 15, and 20 represent cut-off points for mild, moderate, moderately severe and severe depression, respectively (34). The GAD-7 measures anxiety and has seven questions with options ranging from 0 (not at all) to 3 (nearly every day), with an overall scale of 0–21. Scores of 5, 10, and 15 represent cut-off points for mild, moderate, and severe anxiety, respectively (35). Internal reliability of the PHQ-9 and GAD-7 was high and moderate, respectively (α = 0.72 and α = 0.61). As a rule of thumb, Cronbach's alpha of the scale and subscale should be above 0.600 (36, 37).

### Data analysis

All recorded interviews and FGDs were transcribed and translated into English. The study used the thematic data analysis method. Thematic analysis is a method for analyzing qualitative data in many disciplines and fields and can be applied to different datasets to address various research questions—“it also involves interpretation in selecting codes and constructing themes” [(38), P1]. The thematic analysis was conducted using six stages outlined by Braun and Clarke (39). The six stages of the framework include; 1) familiarization with data, 2) generation of initial codes, 3) searching for themes, 4) reviewing themes, 5) defining themes, and 6) analysis and writing up. This method allowed the researchers to closely examine the data to identify common themes—topics, ideas and patterns of meaning that come up repeatedly; it allowed the researchers to analyze the data in five broad steps, namely: familiarization; coding; generating themes; reviewing themes, and defining and naming themes.

Quantitative data entry, cleaning and analysis were done using STATA version 17. Descriptive data are presented as graphs, frequency/percentage distribution and numerical summary tables. In addition, Pearson Chi-square tests were used to explore if depression and anxiety were associated with population characteristics. All analyzes were done at a 95% confidence level.

### Ethical consideration

The study was conducted with complete adherence to ethical standards expressed in the Declaration of Helsinki. Before the commencement of the study, relevant authorization and approval were sought from the University of Malawi Research Ethics Committee (UNIMAREC) (No. P/03/21/53), Mangochi, Blantyre, Lilongwe, and Karonga district commissioners. Permission to conduct the study was also sought from Village and Group Village Heads of areas where data were collected. In addition, a consent form was read to adolescents and parents and guardians of adolescents under 18 who agreed to participate in the study and provided written informed consent to participate in the study.

### Methodological limitations

The study has two methodological limitations. First, although the study has used mixed methods, combining quantitative and qualitative methods, this paper has heavily relied on qualitative data for its analysis and conclusions. Secondly, because we employed convenience sampling techniques, the results may not represent the mental health of adolescents in Malawi.

## Results

The results are discussed in three thematic areas- COVID-19 and Depression, COVID-19 and anxiety, and mental health-related effects of COVID-19.

### Basic demographics and outcomes

As indicated in Table 1, most of the respondents were from Lilongwe (44.1%), whilst the minority were from Karonga (9.7%). There was a similarly equal distribution between males and females. Most participants were primary school-going adolescents (54.9%), and more respondents were over 17 years old (59%).

**Table 1:** Demographics, depression, and anxiety outcomes.

**Variable**	**Total**	**Depression^a^**	**Anxiety^a^**	**Depression**	**Anxiety**
	***N*** **(%)**			**% difference [CI]** ^a^	
**Gender**					
Male	185 (54.4)	41 (22.2)	35 (18.9)	−0.4 [−9.3, 2.9]	−3.7 [−12.3, 5.0]
Female	155 (45.6)	35 (22.6)	35 (22.6)		
**Education level**					
Primary	186 (54.9)	34 (18)	26(14)	−9.2 [−18.2, −0.1]^*^	−14.1 [−22.8, −5.4]^*^
Secondary	153 (45.1)	42 (27)	43 (28)		
**Age**					
< 18 years	139 (41)	28 (20)	22 (16)	−3.7 [−12.6, 5.2]	−8.1 [−16.5, 0.4]
≥18 years	201 (59)	48 (24)	48 (24)		
**District**					
Lilongwe	150 (44.1)	34 (22.7)	31 (20.7)		
Blantyre	91 (26.8)	20 (22.0)	17 (18.7)		
Mangochi	66 (14.4)	15 (22.7)	14 (21.2)		
Karonga	33 (9.7)	7 (21.2)	8 (24.4)		
	Mean (SD) [Min, Max]				
**Age in years**					
Male	17.2 (1.9)	17.6 (1.5)	17.7 (1.3)		
Female	17.1 (1.8)	17.5 (1.4)	17.5 (1.4)		
Total	341 (100)	76 (22.4)	70 (20.6)		

a Proportion with an outcome in a particular category.

b Chi-square test of equality of proportions with an outcome comparing the categories; Male-Female, Primary-Secondary, < 18–≥18 years.

* Significant difference.

#### COVID-19 and depression

**Figure 1:**
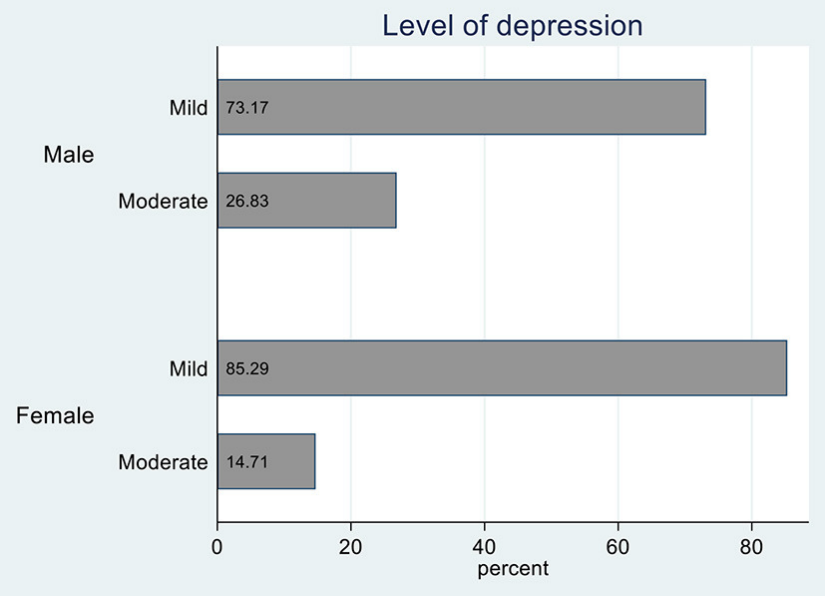
Depression levels segregated by gender.

The results show that approximately 22% of the respondents were depressed. This proportion remained almost constant among various groups of people of different genders and education levels and from other districts (Table 1). Results on depression levels among depressed respondents show that slightly more females (85%) had mild depression than males (73%) (Figure 1). In comparison, more males (26.8%) than females (14.3%) showed moderate depression levels. However, these differences were not statistically significant (Table 1). There was one outlier, one female respondent, who depicted severe depression. The chi-square test results show that significantly more adolescents with secondary education (27%) were depressed than those with primary education (18%).

Multiple factors can explain these results, including school closures, disruption of socioeconomic routines, isolation, and concerns about several factors affecting families, including illness, loss of loved ones and economic impacts. For example, at a focus group discussion in Blantyre, respondents highlighted that COVID-19 had induced fear among family and community members. People had become uncertain about contacting others for fear of contracting or spreading COVID-19. Uncertainties like this and the inability to associate with familiar people may also be linked to anxiety.

The COVID-19 preventive measures counter most cultural and social practices—particularly social distancing and isolation. These measures necessitated the closure of schools, recreation facilities, entertainment places, places of worship and other places where people ordinarily gather. These places allow people to socialize, share experiences and unwind. Thus, one of the recurrent themes that have come up in the findings is anger among the participants. The adolescents reported having developed anger issues due to frustration over the preventive measures that were put in place. Due to the anger, some adolescents committed offenses against the preventative measure, particularly restricting socializing with others. For most adolescents, schools where most socialization happens, new friends are sought and made, but these places were closed, making the adolescents more frustrated. Some adolescents expressed anger toward COVID-19 for disturbing their way of living. During KIIs, some caregivers indicated that “*the adolescents were showing anger, especially when you advise them to observe coronavirus preventive measures such as wearing masks”* (KII Parent).

They also show that respondents had a sense of hopelessness and a lack of interest in social activities due to COVID-19. This led to a loss of hope for the future, as people were unsure if COVID-19 would ever be controlled and returned to their ordinary ways of living. This was expressed during a focus group discussion in Lilongwe: “*Yes, we feel hopeless because we fail to reach our desires regarding our education. Schools are being closed, which makes us doubt that our dreams will ever be fulfilled*” (FGD Lilongwe). Caregivers of adolescents observed that even after relaxing some COVID-19 measures, some adolescents found it challenging to re-adapt. One key informant said: “*Most of the adolescents have not yet returned to school. Most of those that have returned have shown little interest in school”* (KII Head Teacher).

#### COVID-19 and anxiety

**Figure 2:**
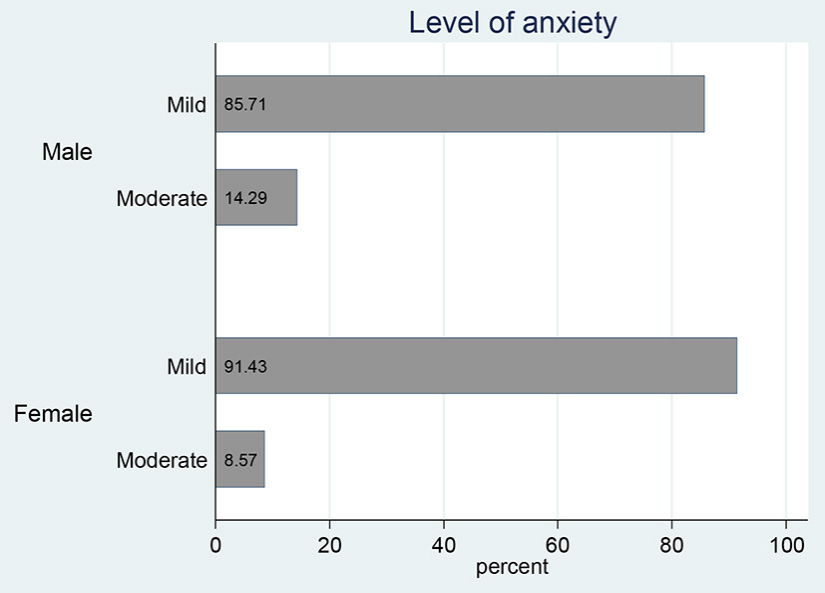
Anxiety levels segregated by gender.

The results indicate that approximately 21% of the respondents had anxiety (Table 1). In contrast to the depression outcome, the proportions of respondents with anxiety varied across different groups. For example, 19% of males and 23% of females reported anxiety symptoms, even though these proportions were not significantly different. The anxiety levels segregated by gender show that 85.7% of males and 91.4% of females had mild anxiety disorder symptoms (Figure 2). To a smaller extent, five males (14.3%) and three females (8.5%) reported moderate anxiety symptoms. The chi-square test results show that significantly more adolescents with secondary education (28%) had anxiety compared with those that had primary education (14%).

Anxiety was observed in the adolescents' statements stating they were no longer confident in their future. They explained that they were not motivated to pursue anything with uncertainties around them because the future was uncertain. Adolescents pointed out persistent rumors that the world would end; hence there was no point in doing anything, including going to school. In Malawi and elsewhere, one of the main motivating factors for people to go to school is to prepare themselves for a better future—education provides life chances more than anything else. One adolescent during a focus group discussion in Karonga said, “*… many people are dying due to this virus, and it has no cure or treatment. So, the thought of coronavirus makes me think that the world is ending”* (FGD Karonga).

Adolescents' anxiety is also manifested through fear. The participants were so fearful of contracting the virus, stating that they were not ready to die. This fear emanated from experience as some lost family members and close friends to the COVID-19 pandemic. These first-hand experiences heightened concerns from the rumors that the world was ending. The stories had cultural resonance, particularly those that people hold dear. For example, participants said there were rumors that those who got the COVID-19 vaccine would never have children. Failure to have children in African communities, especially women, makes one a laughingstock of their community. Culturally, having children is the ultimate goal.

The other fear was the isolation one had to go through if one ever tested positive for COVID-19. They were terrified of being separated from their family members, making them feel like they were not loved, even so, that family members could visit them to cheer them up, as is customary in African cultures. As one focus group participant in Mangochi narrated:

“*When we hear about COVID-19, some people bring up the issue of 666 that is associated with the COVID-19 vaccine. They imply that the world is about to end. So, when people talk about the 666 signs, as a youth, I feel like I have no future, mainly in education. I feel it will be meaningless even if I go to school since the earth will end soon”* (FGD Mangochi).

The adolescents also expressed reduced levels of concentration. Most participants said that they regularly had sleepless nights. This was due to fear of the likelihood of contracting the virus during the day and anxiety about what the next day would bring. This contributed to fatigue, which led lack of concentration the next day and poor performance in various activities. For example, focus group participants in Lilongwe said:

“*There are other times we spent sleepless nights thinking of the coronavirus. When I went to sleep, I would start thinking about my day and all the people I interacted with. This would be worse if I noticed that I coughed at any point. This led to spending the whole night without sometimes sleeping”* (FGD Lilongwe).

We further looked at the relationship between anxiety and depression. The regression results showed that adolescents with anxiety were 18 [95%CI: 9.34, 35.8] times more likely to have depression compared to those who did not have anxiety. The regression model was adjusted for gender, age, education level and district of residence, and none of these variables significantly impacted the outcome.

#### COVID-19, mental health, and related effect

The pandemic has also taken a toll on the education of adolescents. One of the teachers expressed concern about the pandemic's adverse effects on adolescents' schooling. In her explanation, she said;

“*COVID-19 pandemic has caused many problems in the lives of many youths. For instance, due to the school closure, many students, especially the standard eight female students who registered to write their final exams, dropped out of school because they were pregnant. On top of that, even after the schools were opened, many students had little interest in school, resulting in poor performance in examinations”* (KII Parent).

One of the key informants expressed concern that the pandemic made the students consider school useless. This was because schools kept being closed and re-opened. This view is reflected by one of the adolescents who said the following after being asked how the pandemic had affected her: *My life changed when I started hearing about COVID-19. First, my education was affected because I lost interest and stopped working hard (FGD 2)*.

When asked how COVID-19 had affected the lives of adolescents, they expressed their frustration with how much damage COVID-19 did financially. One respondent said:

“*With the measures put in place, life has not been the same; we are restricted from public gatherings, and businesses are not operating as they used to. This has also impacted our parents financially. They are having difficulties paying our school fees”* (FGD Karonga).

As presented above, anxiety, depression and stress were commonly reported experiences among adolescents during COVID-19. However, participants expressed that none of them sought mental health help. The headmasters, teachers, and guardians interviewed also said that they had not offered mental health guidance to adolescents under their care, as expressed by a headteacher in Lilongwe; “We must be honest; we do not have any mental health programs set aside.” Further, they expressed a lack of knowledge on identifying mental health cases. Through experience, they notice mental health cases; however, they claim to have received no formal or systematic training on mental health.

The lack of seeking mental health services may also be related to the community's perception of mental health. A faith leader expressed that many communities in this country still do not take mental health issues seriously. He stressed that mental health patients are often ignored because mental health issues are not regarded as problems. When asked why this was the case, the Faith Leader (Karonga) explained that “a person ill with other diseases can easily be recognized by the symptoms they show, unlike a mental health patient.” He further said that mental health issues are hard to be recognized by an ordinary person. One other reason associated with low mental health services utilization is stigmatization. A parent in Mangochi explained that “because of stigmatization, people may not want their mental health issues to be made known to others.”

## Discussion

The paper examined the effects of the COVID-19 pandemic on the mental health of adolescents in Malawi. The results show that many adolescents in Malawi experience signs and symptoms of mental health problems or disorders, mostly without knowing it. The lack of knowledge means that those affected could not seek help. The lack of adequate knowledge correlates with a study by Crabb et al. (40), which established a knowledge gap on mental health issues among Malawians. The lack of knowledge worsens the problems as it negatively affects help-seeking behavior among adolescents experiencing mental health problems in the country.

The lack of knowledge may emanate from the fact that mental illness is not openly discussed In Malawi, in the same way as other illnesses, such as Tuberculosis, which means most people lack basic knowledge about mental illness. The lack of knowledge leads to misconceptions, falsehoods and misinformation about mental health, which may lead to stigma and discrimination against people with mental illness. For instance, if one is unaware of the causes of mental health problems and that mental health matters the same way physical health does, one may feel isolated and misunderstood by society. Furthermore, people with wrong perceptions and beliefs about people with mental illnesses tend to judge and treat them from a place of ignorance. As Crabb et al. (40) discussed, these are some of the reasons many cases of mental illnesses in Sub-Saharan Africa are treated are either ignored or treated punitively.

The study also indicates that a good percentage of adolescents experienced anxiety. However, when anxiety is left untreated, it can have long-term consequences that can lead to impairment in daily life. Anxiety disorder can manifest in different ways, including; poor performance in school, behavioral problems, dropping out of school, poor self-worth, low self-esteem, teen pregnancy, missing out on social engagements, substance use, and abuse. Comparatively, females showed a higher percentage of anxiety symptoms than males. This is consistent with several studies on gender differences in different anxiety disorders. According to Hou et al. (41), females suffer from anxiety more than males and are diagnosed with the most anxiety disorders. Studies have consistently shown that one of the factors contributing to this is that males have better resilience to stress than females.

Another way COVID-19 may have affected adolescents' mental health was through depression. Just like the case of anxiety, slightly more females showed symptoms of depression than males. This may be because, in African societies, females are the primary caregivers and home keepers and are at greater risk for psychological problems than their male counterparts (41). They found that the overall prevalence of mental disorders was 5.9% and was higher in males (7.1%) than in females (4.7%). Hence, more research is needed in sub-Saharan Africa to understand the scale of adolescent mental health problems (12). In addition, untreated depression may lead to adverse effects. People who suffer from depression may end up experiencing low self-esteem, alcoholism, substance abuse, academic problems, difficulty maintaining relationships, social isolation, self-harming behaviors, and Suicide. Another study on a Chinese population during the COVID-19 epidemic showed more depression and severe anxiety symptoms in females than males (41). However, these results are contrary to Matandika et al. (12). Matandika et al. (12) found that the prevalence of mental disorders was higher in males than in females. Hence, more research is needed in sub-Saharan Africa to understand the scale of adolescent mental health problems. These contrary results may be attributed to a difference in measurement tools in that Matandika et al. used a general tool for assessing mental health disorders and demographic characteristics of the sample in that Matandika's sample was primarily urban.

Recent studies have also shown that COVID-19 affect mental health outcomes such as anxiety, depression, and post-traumatic stress symptoms (42). One of the adverse effects of anxiety is that when it is above average, it weakens the body's immune system and consequently increases the risk of contracting the virus (6). Misinformation and flooding of COVID-19-related information on social media contributed to anxiety among adolescents. Information shared among adolescents during the pandemic may have contributed significantly to the anxiety they experienced. One common misinformation was that the world was coming to an end. Adolescents believed that COVID-19 was one of the signs that the world was coming to an end, hence. They experienced anxiety in different ways; whilst some adolescents experienced a lack of concentration, others were constantly afraid that something terrible would happen to them or their families, and yet others were apprehensive when someone they knew tested positive for COVID-19. The misinformation that the world is ending has resonance beyond COVID-19. Malawi is a hugely Christian nation in which most people are familiar with biblical tales such as the last days and the scourge of diseases that will appear on planet earth. This makes it easy for people to buy into misinformation about the world ending. Misinformation always takes cultural resonance, which is why it varies worldwide.

The pandemic took a toll on the education of adolescents as well. During the pandemic, young people lost interest in concentrating on their studies. Some believed that the world was ending, others anticipated that school would keep being open and closed, whilst others decided to invest their interest in other things like marriage or being pregnant. The COVID-19 pandemic worsened many pre-existing crises, including an education emergency that has resulted in high out-of-school rates, particularly among adolescents and young people. Despite Malawi not being under total national lockdown, young people experienced financial setbacks because schools were closed and social gatherings reduced to small numbers, and there was less time for interaction. Adolescents' most significant concerns during the COVID-19 crisis were disrupting their social interactions, losing their loved ones, contracting the virus, and not being able to complete their education and others.

All in all, the measures taken to ensure that the spread of the virus was reduced were the ones that caused more distress than the disease itself. Interestingly, results showed that significantly more secondary school adolescents were depressed than primary school adolescents. This may be explained by two contextual factors, knowledge and responsibility. First, higher education level is associated with comprehension. So, secondary school adolescents may have understood the dangers of COVID-19 more than primary school adolescents, which may have led to more depressive symptoms. Secondly, as age comes with more responsibilities in Africa, secondary school adolescents may have assumed different duties from the burden of the pandemic, hence more depressive symptoms.

The current study found a relationship between those who were depressed and those who had anxiety. This result is not surprising as research has shown that these mental health problems are very related and form comorbidities of each other. For instance, a study by Nwafor et al. (10) found high levels of depression (45.2%) and anxiety (37.5%) among pregnant women. Further, the study suspected that the prevalence of such disorders might have been aggravated during the coronavirus disease 2019 (COVID-19) pandemic because pregnant women may have experienced restricted access to mental health services (10).

The current study has shown that COVID-19 may have contributed to the mental health state of adolescents. This may be because adolescence is a transitional physical and psychological development stage, with social connection playing a fundamental role. To contain the spread of COVID-19, requirements like physical distancing were put in place. Unfortunately, this might have contributed to the absence of many regular sources of social connection in people's lives. The lack of these positive reinforcements may have caused adolescents to experience mood swings, over-eating etc., which are symptoms of depression and anxiety. Nevertheless, the COVID-19 pandemic may have contributed positively to adolescent mental health; during and after the pandemic, more adolescents have talked about mental health issues more freely than before (43). In addition, there has been a rise in mental health awareness campaigns and advocacy, and most importantly, many adolescents are now seeking mental health professional help.

## Conclusion

Although adolescents are often overlooked in the COVID-19 discourse, as COVID-19 significantly affects older people, this study has found that COVID-19 involved adolescents as much as it affected the adult population. COVID-19 may have contributed to the mental health of adolescents in Malawi, although most of them were probably unaware of the signs and symptoms of depression and anxiety that they had. These signs emanated from COVID-19 preventive measures, especially social distancing and self-isolation, introduced by the Government to contain and control the spread of COVID-19. Although Malawi did not institute a national lockdown, unlike most African countries, self-isolation and social distancing inevitably led to the closure of schools and recreation facilities, which are familiar sources of social connection for adolescents. Further, these measures affected economic income leading to worries about survival and attainment of basic needs. Adolescents, although not homeowners, were equally affected by seeing their parents struggling to provide for them. The study's results also highlight contextual factors and experiences of adolescents during COVID-19 and how that affected their mental health. This study's findings significantly contribute to the current research on mental health among adolescents in Africa and Malawi. There are limited data and statistics on mental health and mental disorders in Africa; this study has contributed to the availability of mental health-related data.

## Data availability statement

The raw data supporting the conclusions of this article will be made available by the authors, without undue reservation.

## Ethics statement

The studies involving human participants were reviewed and approved by University of Malawi Research Ethics Committee (UNIMAREC). Written informed consent to participate in this study was provided by the participants' legal guardian/next of kin.

## Author contributions

CM, YN, JK, SN, FK, MM, and LM conceptualized the idea and reviewed the manuscript. CM, YN, and FK analyzed the data. CM, YN, and JK drafted the paper. All authors contributed to the article and approved the submitted version.
